# The role of P2X7 receptors in a rodent PCP-induced schizophrenia model

**DOI:** 10.1038/srep36680

**Published:** 2016-11-08

**Authors:** Bence Koványi, Cecilia Csölle, Stefano Calovi, Adrienn Hanuska, Erzsébet Kató, László Köles, Anindya Bhattacharya, József Haller, Beáta Sperlágh

**Affiliations:** 1 Laboratory of Molecular Pharmacology, Institute of Experimental Medicine, Hungarian Academy of Sciences (IEM HAS), H-1450 Budapest, Hungary; 2 János Szentágothai School of Neurosciences, Semmelweis University School of PhD Studies, Budapest, Hungary; 3 Department of Pharmacology and Pharmacotherapy, Faculty of Medicine, Semmelweis University, H-1089 Budapest, Hungary; 4 Janssen Research & Development, LLC, Neuroscience TA, San Diego, CA 92121, USA; 5 Department of Behavioral Neurobiology, Institute of Experimental Medicine, Hungarian Academy of Sciences (IEM HAS), H-1450, Budapest, Hungary

## Abstract

P2X7 receptors (P2X7Rs) are ligand-gated ion channels sensitive to extracellular ATP. Here we examined for the first time the role of P2X7R in an animal model of schizophrenia. Using the PCP induced schizophrenia model we show that both genetic deletion and pharmacological inhibition of P2X7Rs alleviate schizophrenia-like behavioral alterations. In P2rx7+/+ mice, PCP induced hyperlocomotion, stereotype behavior, ataxia and social withdrawal. In P2X7 receptor deficient mice (P2rx7−/−), the social interactions were increased, whereas the PCP induced hyperlocomotion and stereotype behavior were alleviated. The selective P2X7 receptor antagonist JNJ-47965567 partly replicated the effect of gene deficiency on PCP-induced behavioral changes and counteracted PCP-induced social withdrawal. We also show that PCP treatment upregulates and increases the functional responsiveness of P2X7Rs in the prefrontal cortex of young adult animals. The amplitude of NMDA evoked currents recorded from layer V pyramidal neurons of cortical slices were slightly decreased by both genetic deletion of P2rx7 and by JNJ-47965567. PCP induced alterations in mRNA expression encoding schizophrenia-related genes, such as NR2A, NR2B, neuregulin 1, NR1 and GABA α1 subunit were absent in the PFC of young adult P2rx7−/− animals. Our findings point to P2X7R as a potential therapeutic target in schizophrenia.

The homo-oligomeric P2X7 receptor (P2X7R) belongs to the ionotropic P2X receptors that are sensitive to ATP and other purine and pyrimidine nucleotides^1^,^2^. P2X7R mediated signaling has received considerable attention in the past years as a signaling highway of immune response, cell survival and CNS pathology^3^,^4^. P2X7R activation provides the co-stimulus for the posttranslational processing of the pro-inflammatory cytokine IL-1β through the activation of the NLRP3 inflammasome, and also regulates neurotransmitter release and neuronal survival in the CNS^5^.

In rodent studies, both genetic deletion and pharmacological inhibition of P2X7Rs lead to an antidepressant phenotype in behavioral paradigms mimicking depressive-like behavior^6^,^7^,^8^,^9^. In addition, the same manipulations attenuate amphetamine induced hyperactivity mimicking the manic pole of bipolar disorder^7^,^10^,^11^,^12^. These findings indicate that endogenous activation of P2X7Rs contributes to behavioral changes induced by either negative or positive challenges, and the blockade of the receptor alleviates these fluctuations. Therefore P2X7Rs have been suggested as a potential target to treat mood disorders^4^,^5^,^13^.

Emerging data also suggest that central P2X7Rs are involved in the regulation of other complex behaviors, such as cognition and social activity. Mice deficient in P2rx7s display spatial memory impairment in a hippocampal-dependent memory task coincidently with a decreased induction of IL-1β and c-fos in the hippocampus^14^. However, the selective P2X7R antagonist, A-740003 interacts with acquisition, consolidation and retrieval of aversive memory, whereas habituation memory is not affected^15^. Further, JNJ-42253432, a brain-penetrant P2X7R antagonist increases overall social activity and social preference^11^. Therefore, P2X7R modulation may represent an intriguing possibility for interfering with neuropsychiatric conditions characterized by cognitive and social deficit (e.g. schizophrenia, autism). Another indication for the role of P2X7R in psychoses comes from studies showing that the phenothiazine-class antipsychotic drugs are potent allosteric modulators of the human P2X7R^16^.

In the hippocampus, P2X7R immunoreactivity is localized to the membrane of excitatory nerve terminals synapsing onto parvalbumin positive postsynaptic GABAergic interneuron targets, and activation of P2X7Rs elicits glutamate and subsequent GABA release^17^,^18^. P2X7R activation releases glutamate from cerebrocortical nerve terminals as well^19^,^20^.

Morphological and functional alteration of the microcircuits in the dorsolateral prefrontal cortex (PFC) during development is thought to be a core feature of schizophrenia^21^. A prominent feature of these circuitry alterations is the reduced excitatory input to layer 3 pyramidal neurons of the PFC and subsequent NMDA receptor hypofunction, as well as the consequently reduced transmission in the parvalbumin positive GABAergic interneurons^22^,^23^. The resultant decrease in GABA signaling to PFC pyramidal neurons can lead to hyperexcitability of pyramidal neurons and might contribute to reduced γ-oscillations and subsequent impairment of working memory performance in schizophrenic patients. Therefore, molecular signaling entities targeting relatively selectively NMDA receptor hypofunction and its consequences could alleviate the symptoms of schizophrenia.

NMDA receptor hypofunction is mimicked by the psychostimulant phencyclidine (PCP) in animal experiments, which reproduce both positive and negative symptoms of schizophrenia^24^. Because it is known P2X7R activation influences cortical glutamatergic transmission, it was worthwhile to examine the role of this receptor in PCP induced schizophrenia model in mice. In addition to behavioral changes, as a potential mediator of P2X7R induced changes, glutamatergic transmission was examined in acute prefrontal cortical slices. Finally, gene expression changes were also mapped in the hippocampus and prefrontal cortex in juvenile and young adult animals.

## Results

### Effect of genetic deletion and pharmacological inhibition of P2X7R on PCP induced behavioral alterations

**Figure 1:**
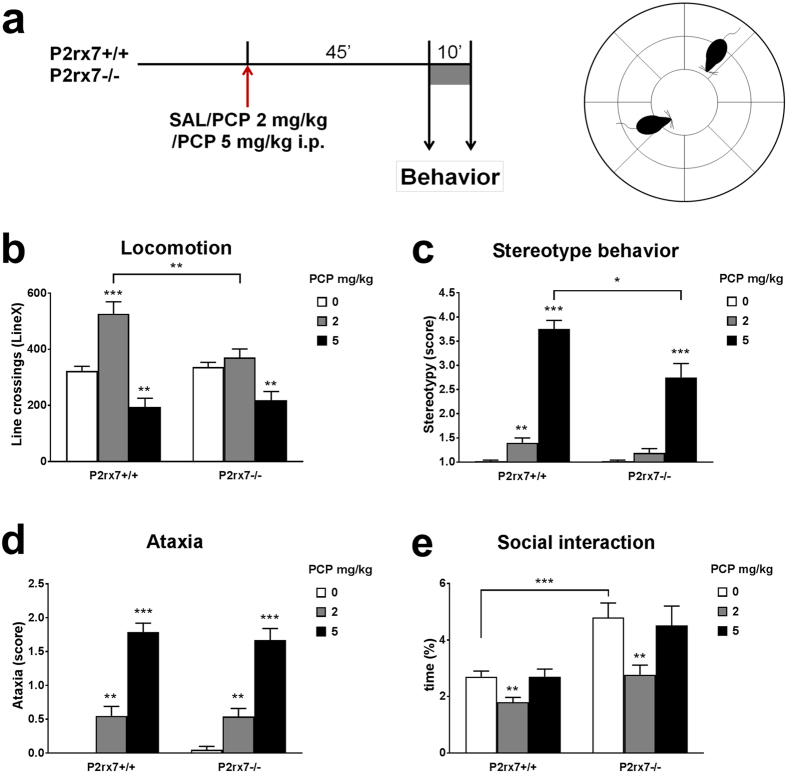
Effect of P2rx7 gene deficiency on PCP-induced behavioral changes in mice. (**a**) Drug and test naïve C57Bl/6J P2rx7+/+ and P2rx7−/− mice were injected with PCP (2 or 5 mg/kg i.p.) or saline and behavior were recorded 45 min later, during a 10 min test period, as described in the methods. (**b–e**) Different aspects of PCP induced behavioral changes, i.e. hyperlocomotion, stereotype behavior, ataxia and the time of social interactions were quantified offline by an experimenter blind to the treatments. Asterisks indicate significant changes between genotypes or vs. saline treated group, as indicated (*p < 0.05, **p < 0.01, ***p < 0.01). Data were analyzed by Kruskal-Wallis ANOVA. n = 10–14/group.

At first, we examined, how P2rx7 gene deficiency affect PCP induced changes in behavior (Fig. 1a). P2rx7+/+ and P2rx7−/− mice were treated with two different doses of PCP (2 or 5 mg/kg i.p.) or its vehicle (saline, SAL) and different aspects of behavior analyzed offline during a 10 min trial period applied 45 min after the PCP/SAL treatment (Fig. 1a). PCP treatment significantly affected all four aspects of behavior, i.e. locomotion, stereotypy, ataxia, and social interactions (H(5, n = 95)) values were 37.47, 77.09, 78.58, and 32.03, respectively; p < 0.0001 in all cases). Basal levels of locomotion, stereotypy and ataxia were not affected by genotype, but the duration of social interactions was significantly higher in P2rx7−/− as compared with P2rx7+/+ controls. In controls, PCP exerted its well-known effects: increased locomotion, stereotypy and ataxia, and decreased social interactions. The effect was dose dependent in the case of stereotypy and ataxia, and biphasic in the case of the other two variables. The decrease of locomotion at the higher dose of PCP was due to the parallel increase in ataxia and stereotypy, which prevented mice from covering large distances. Social interactions on their turn were normalized by the increase in circling behavior, the typical stereotypic behavior displayed at 5 mg/kg PCP (stereotypy score 4). Mice often circled close to each other, for which their noses often touched the body of their partners; moreover, they sniffed at each other briefly. The effects of PCP were milder in the P2rx7−/− genotype. The lower dose did not increase locomotion (Fig. 1b); albeit the higher dose decreased it, this was due to high ataxia scores, which were not affected by gene disruption (Fig. 1d). The 2 mg/kg dose did not increase stereotypy significantly, whereas the larger dose induced a smaller increase than in wild-types (Fig. 1c). The effects of PCP on social interactions were similar in the two genotypes, except that higher values were observed overall in knockouts (Fig. 1e).

**Figure 2:**
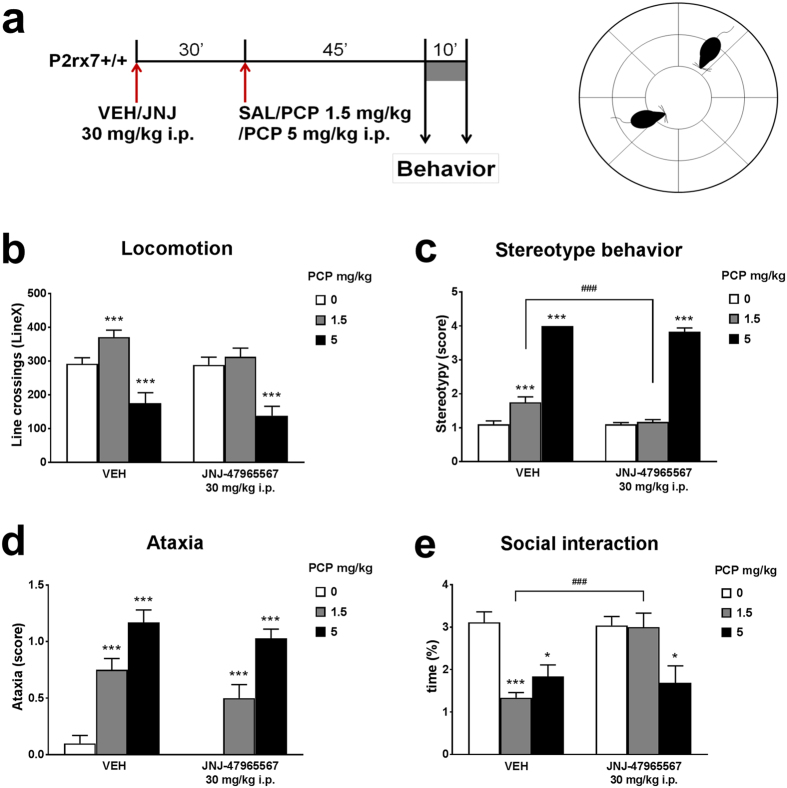
Effect of the selective P2X7R antagonist JNJ-47965567 (30 mg/kg i.p.) on PCP-induced behavioral changes in mice. (**a**) Drug and test naïve C57Bl/6J P2rx7+/+ and P2rx7−/− mice were injected with JNJ-47965567 (30 mg/kg i.p.) or its vehicle 30 min prior to subsequent PCP (1.5 or 5 mg/kg i.p.)/saline injections. Behavior was recorded 45 min later, during a 10 min test period, as described in the methods. (**b–e**) Different aspects of PCP induced behavioral changes, i.e. hyperlocomotion, stereotype behavior, ataxia and the time of social interactions were quantified offline by an experimenter blind to the treatments. Symbols indicate significant changes (***p < 0.001 vs. SAL, ^###^p < 0.001, vs. VEH). Data were analyzed by Kruskal-Wallis ANOVA. n = 10-12/group.

Next, we have attempted to replicate findings obtained with P2rx7 gene deficient mice by systemic treatment with the selective, brain permeable P2X7 receptor antagonist JNJ-47965567 (Bhattacharya *et al*.^10^), which was administered as a single i.p. treatment at a 30 mg/kg dose 30 min prior to PCP regimen (Fig. 2a). Again the typical effects of PCP were observed (H(5, n = 74)) values were 32.26, 53.20, 51.01, and 28.34 for locomotion, stereotypy, ataxia, and social interactions, respectively; p < 0.0001 in all cases). There was no effect of the vehicle on PCP-induced behavioral changes; in-fact the vehicle + PCP group in this study was similar to P2rx7+/+ controls from the previous experiment shown in Fig. 1. (Figure 2b–e). The antagonist per se (i.e. without PCP) had no significant effect on any of the behaviors; by contrast, the effects of the lower dose (1.5 mg/kg) of PCP were abolished except for ataxia: locomotion, stereotypy and social behaviors were not affected by the low dose of PCP in mice pre-treated with JNJ-47965567 (Fig. 2b,c,e). JNJ-47965567 failed to modulate any of the behaviors manifested by high dose (5 mg/kg) of PCP.

### Developmental changes of P2rx7 mRNA expression in the mouse prefrontal cortex and hippocampus

**Figure 3:**
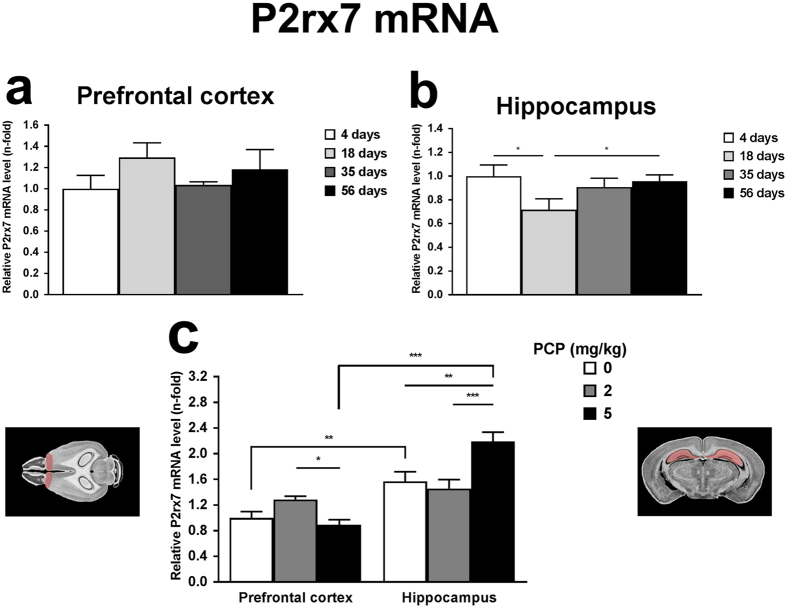
Age-dependence and effect of PCP on P2rx7 mRNA expression in the prefrontal cortex and hippocampus. Developmental changes of P2rx7 relative mRNA expression in prefrontal cortex (**a**) and hippocampus (**b**) of naïve C57Bl/6J P2rx7+/+ mice of four age groups (4, 18, 35 and 56 days). Gene expression levels of P2rx7 were normalized to the gene expression level of Gapdh, as the endogenous control gene. The normalized P2rx7 mRNA expression levels of 4 days old animals were interpreted as 100% to compare the P2rx7 gene expression level in distinct age groups. Data are displayed as the mean ± S.E.M. (*p < 0.05, one-way ANOVA followed by Fisher’s LSD post hoc test). n = 6–8/group. (**c**) Effect of PCP on P2rx7 mRNA expression levels in the mouse prefrontal cortex and hippocampus. Young adult (56 days old) animals were given an intraperitoneal (i.p.) injection of sterile saline (0.9% NaCl) or PCP in two distinct doses (2 and 5 mg/kg). Gene expression levels of P2rx7 were normalized to the gene expression level of Gapdh, as the endogenous control gene. The normalized P2rx7 mRNA expression level in the PFC of the saline-treated group was interpreted as 100%. Data are displayed as the mean ± S.E.M. (*p < 0.05, **p < 0.01, ***p < 0.001; two-way ANOVA followed by Fisher’s LSD post hoc test). n = 3–6/group. Illustrations of coronal section 21 and horizontal section 4 of the mouse brain are originated from the website of http://www.mbl.org (PFC and HPC regions are highlighted)^69^.

In the next set of experiments, real-time PCR analysis was used to measure the relative P2X7 receptor mRNA levels in four age groups of naïve P2rx7+/+ mice (4, 18, 35 and 56 days) in order to reveal any developmental regulation of its expression in two brain regions implicated in PCP-induced behavioral changes, i.e. the prefrontal cortex and hippocampus. Data were normalized to the group of 4 days old mice (Fig. 3a,b). There was no statistical significant difference among the distinct age groups in prefrontal cortex tissues (Fig. 3a). In contrast, the P2X7 receptor mRNA levels in hippocampal tissues was significantly decreased in the group of 18 days old mice (0.72 ± 0.09) compared to the group of 4 days old mice (1.00 ± 0.09, n = 6–8, p < 0.05). This decline is normalized during the adolescence and P2rx7 mRNA levels detected in 56 says old, young adult animals were similar to the 4-days old group (0.957 ± 0.05, n = 7) (Fig. 3b).

### Effect of PCP on P2rx7 mRNA expression in the mouse prefrontal cortex and hippocampus

Next, we have addressed the question, whether PCP treatment induce any change of P2rx7 mRNA expression in the prefrontal cortex and hippocampus. Discrepancies in relative mRNA expression levels of P2X7 receptor were also investigated in prefrontal cortex and hippocampus samples of naïve, young adult (56 days) P2rx7+/+ mice after three distinct treatments, i.e. sterile saline (0.9% NaCl) or PCP in two different dosages (2 or 5 mg/kg) (Fig. 3c). All data of normalized P2rx7 gene expression levels were compared to the group of prefrontal cortex after saline injection (1.00 ± 0.10). PCP treatment elicited region- and dose-related alterations in P2X7 receptor mRNA expression in each brain regions. In the prefrontal cortex, the lower, 2 mg/kg dose of PCP slightly elevated (1.29 ± 0.05, p = 0.07) P2X7 receptor mRNA level, whilst the higher, 5 mg/kg dose did not change the expression (0.89 ± 0.08, n = 3, p > 0.05). In contrast, in the hippocampus, 5 mg/kg dose of PCP lead to statistically significant upregulation (2.19 ± 0.1) in the gene expression level of P2rx7 compared to saline (1.57 ± 0.15, n = 4, p < 0.01), whereas the 2 mg/kg dose of PCP was without effect (1.46 ± 0.13, n = 6, p > 0.05). Interestingly, in saline treated groups (PFC: 1.00 ± 0.10, HPC: 1.57 ± 0.15, n = 5–7, p < 0.01), and in the groups treated by the higher PCP dosage (PFC: 0.89 ± 0.08, HPC: 2.19 ± 0.14, n = 3–4, p < 0.001) hippocampal P2X7 receptor mRNA level was significantly higher than in the prefrontal cortex.

### P2X7R mediated [^3^H]glutamate ([3H]Glu) release is upregulated after PCP treatment in the prefrontal cortex

**Figure 4:**
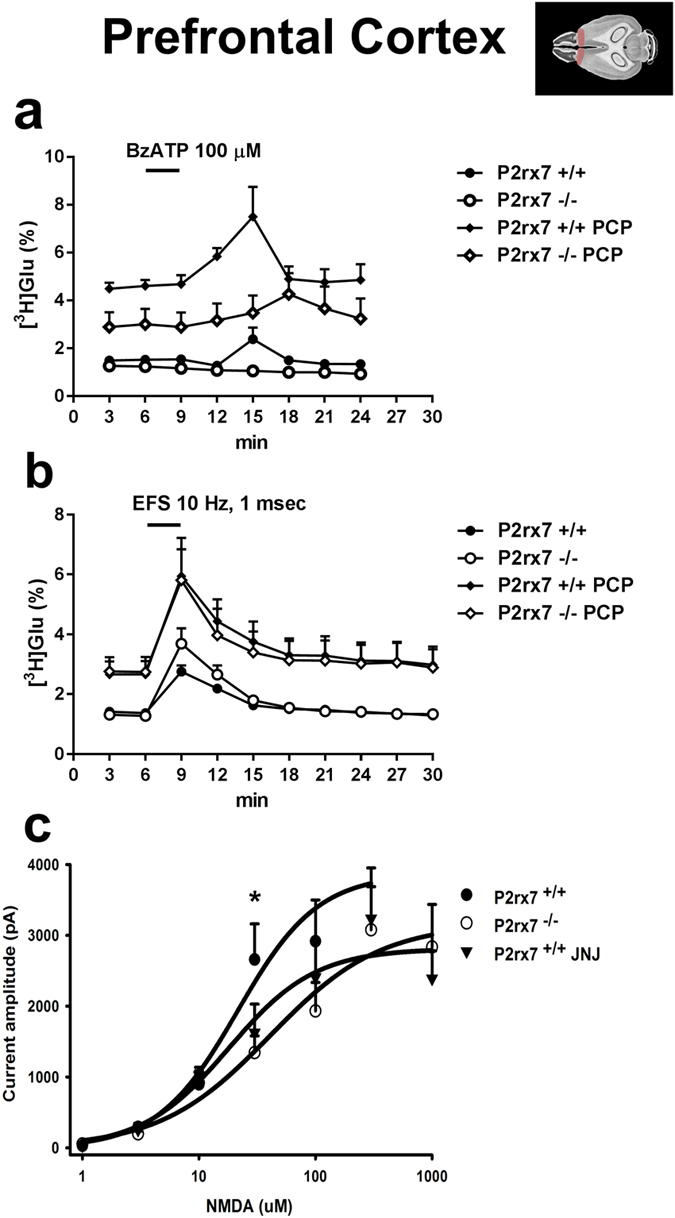
Modulation of the glutamatergic transmission in the mouse prefrontal cortex by P2X7 receptors. Effect of lower dose of PCP treatment (2 mg/kg i.p.) on P2X7R mediated (**a**) and electrically evoked (**b**) [^3^H]glutamate release from the *ex vivo* acute PFC slices. C57Bl/6J P2rx7+/+ and P2rx7−/− mice were injected with PCP (2 mg/kg i.p.) or saline and acute PFC slices were prepared 60 min later and incubated with [^3^H]glutamate (see Methods). During the sample collection period, slices were stimulated by the P2X7R agonist BzATP (100 μM) or electrical field stimulation (EFS), as indicated by the horizontal bars. Data are displayed as the mean ± S.E.M. n = 8-12/group. (**c**) Concentration-response curves of NMDA in P2rx7+/+ mice in the presence or in the absence of the P2X7R antagonist JNJ-47965567 (100 nM, JNJ) as well as in P2rx7−/− mice. NMDA (1-1000 μM) was applied in increasing concentrations and the amplitude of the NMDA evoked inward currents were recorded from layer V pyramidal neurons of cortical slices. Concentration-response curves for NMDA were fitted using the logistic function of SigmaPlot (Systat). Data are displayed as the mean ± S.E.M. of n = 5-16 cells at different NMDA concentrations (*p < 0.05; P2rx7−/− vs. P2rx7+/+; two-way ANOVA). Illustration of horizontal section 4 of the mouse brain is originated from the website of http://www.mbl.org (PFC region is highlighted)^69^.

Because the lower dose of PCP treatment (2 mg/kg i.p.) upregulated P2rx7 mRNA expression in the prefrontal cortex, it was worthwhile to examine, whether functional responsiveness of P2X7 receptors are also subject to regulation by PCP in this brain region. P2X7 receptor activation is known to release glutamate from cortical nerve terminals, therefore, glutamate efflux induced by the P2X7 preferring agonist BzATP from acute prefrontal cortex slices was used as a probe of P2X7R activation and its effect was compared to that of electrical field stimulation (EFS) in *ex vivo* saline and PCP treated wild-type and P2rx7 deficient animals, respectively. After loading the prefrontocortical slices with [^3^H]Glu and 90 min preperfusion, the uptake of radioactivity was 513.9 ± 28.9 kBq/g (n = 8) in saline treated P2rx7+/+ mice, and 857.9 ± 10.06 kBq/g (n = 12, p < 0.05) in the P2rx7−/− mice. The basal efflux of [^3^H]Glu, was 1.58 ± 0.06% (n = 8) and 1.26 ± 0.05% (n = 12, p > 0.05) in P2rx7+/+ and P2rx7−/− mice, respectively, not significantly different from each other. A 3-min perfusion with the P2X7R agonist, BzATP (100 μM) elicited a transient elevation in the efflux of [^3^H]Glu in P2rx7+/+ mice, which was reversible upon washout (Fig. 4a). No increase in tritium efflux was detected in response to P2X7R agonist in the slices of P2rx7−/− mice (Fig. 4a). When the slices (wild-type and knockout) were treated with PCP for 60 min, there was enhanced release of [^3^H]Glu as shown in Fig. 4a. Preceding PCP treatment did not change radioactivity uptake into the slices in P2rx7+/+ mice (363.33 ± 86.5 kBq/g, n = 8, p > 0.05 vs SAL). The basal release was higher in P2rx7+/+ slices than in P2rx7−/− slices. BzATP induced tritium efflux was enhanced after PCP treatment in P2rx7+/+ mice (evoked release: 1.5 ± 0.33%, n = 8 and 4.18 ± 0.91% in saline and PCP treated mice, n = 8 each, p < 0.05 (Fig. 4a)).

Electrical field stimulation also elicited reversible [^3^H]Glu efflux in P2rx7+/+ mice, which was not decreased in P2rx7 deficient mice (Fig 4b). EFS induced [^3^H]Glu efflux was also enhanced by PCP in P2rx7+/+ animals (Fig. 4b, evoked release: 1.5 ± 0.57% and 7.42 ± 1.39% in saline and PCP treated mice, n = 8 each, p < 0.01). EFS increased Glu efflux in PCP-treated P2X7−/− mice, when compared to baseline (Fig. 4b). However, the net EFS evoked Glu efflux in these animals was not significantly different from saline treated P2rx7 −/− animals (Fig. 4b, evoked release: 4.73 ± 1.0%, n = 12 and 5.72 ± 2.38% n = 8, in saline and PCP treated mice, p > 0.05).

To explore any effect on dopaminergic transmission [^3^H]dopamine ([^3^H]DA) release experiments were also performed. In these experiments, the radioactivity uptake was 251.144 ± 17.429 kBq/g and 279.789 ± 20.905 kBq/g, (n = 14; 6, p > 0.05), whereas the resting [^3^H]DA efflux was 1.23 ± 0.05% and 0.99 ± 0.03% in P2rx7+/+ and P2rx7−/− mice, respectively (p < 0.001). Perfusion of the slices with ATP (10 mM) elicited a transient elevation of tritium efflux, but this was not affected by the genetic deletion of P2X7 receptors (evoked release: 1.24 ± 0.22%, n = 12 and 1.01 ± 0.12% n = 6, in P2rx7+/+ and P2rx7−/− mice, p > 0.05).

### Effect of genetic deletion and pharmacological inhibition of P2X7R on NMDA induced currents recorded from the mouse prefrontal cortex

The previous experiments clarified that PCP treatment upregulates mRNA level and increase the functional responsiveness of P2X7 receptors in the prefrontal cortex, resulting in a higher elevation of glutamate efflux. Next, we examined, whether endogenous P2X7 receptor activation alter the responsiveness of prefrontocortical NMDA-type glutamate receptors using the whole cell patch clamp technique. Application of various concentrations (1–1000 μM) of NMDA to layer V pyramidal cells of prefrontal cortex induced inward current responses both in P2rx7+/+ and P2rx7−/− mice (n = 5–16 at different concentrations). It is noteworthy, that the pyramidal cells mostly showed large biological variability in sensitivity to NMDA making difficult the statistical analysis.

At low NMDA concentrations (1–10 μM) notable difference between the responses to NMDA in cells derived either from P2rx7+/+ or from P2rx7−/− mice could not be observed (Fig. 4c). However, when higher agonist concentration was applied (30 μM NMDA), a significant difference was revealed in the amplitude of the NMDA currents, i.e. the responses were larger in wild-type animals (2658 ± 504 pA) compared with those lacking P2X7 receptors (1343 ± 238 pA). The concentration-response curves indicated similar tendency at still higher NMDA concentrations resulting in higher E_max_ value of the curve in wild-type mice compared with the P2rx7−/− animals (4330 ± 1171 pA vs 3224 ± 693 pA). Other major parameters of the concentration-response curves (EC_50_: 24.98 ± 0.27 vs. 41.78 ± 0.26; Hill coefficient: 0.91 ± 0.58 vs. 0.85 ± 0.57; wild type vs. P2rx7−/− mice, respectively) did not display any remarkable difference.

Subsequently, the NMDA concentration-response relationship was also investigated in layer V pyramidal cells of the wild-type mice in the presence of the P2X7 antagonist JNJ-47965567 (0.1 μM). As Fig. 4c shows, the pharmacological blockade of the P2X7 receptors apparently had similar influence on the curve like the genetic deletion of the receptor. The main observation is that the E_max_ of the curve in the presence of the P2X7 antagonist was reduced (2831 ± 360 pA) compared with the curve in the absence of the antagonist (see above). However, the clear tendency did not result in a statistically significant difference.

### Genetic deletion of P2rx7 causes region specific changes in the expression of glutamate and GABA receptor subunits and schizophrenia related genes

**Figure 5:**
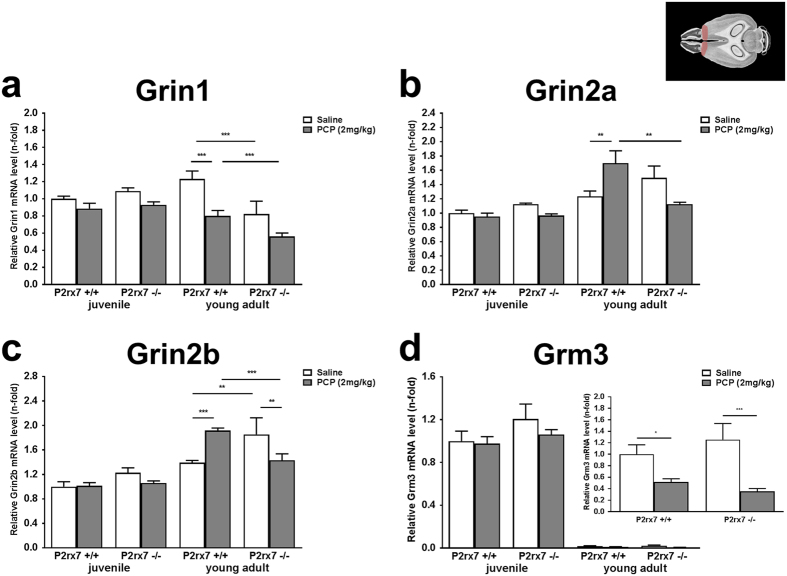
Effect of the lower dose of PCP (2 mg/kg) on the relative mRNA expression levels of ionotropic NMDA, (**a**) Grin1, (**b**) Grin2a, (**c**) Grin2b and a metabotropic glutamate receptor, (**d**) Grm3. Samples are derived from PFC of juvenile (18 days old) and young adult (56 days old), P2rx7+/+ and P2rx7−/− mice. Gene expression levels of each gene were normalized to the gene expression level of Gapdh. Normalized mRNA expression levels in juvenile, saline-treated P2rx7+/+ animals were interpreted as 100%. Data are displayed as the mean ± S.E.M. (*p < 0.05, **p < 0.01, ***p < 0.001; Two-way ANOVA followed by Fisher’s LSD post hoc test). (d inset) Relative mRNA expression levels of metabotropic (Grm3) glutamate receptor in PFC of young adult (56 days old), P2rx7+/+ and P2rx7−/− mice. Gene expression levels of Grm3 was normalized to the gene expression level of Gapdh. Normalized mRNA expression levels in the group of saline-treated P2rx7+/+ animals were interpreted as 100%. Data are displayed as the mean ± S.E.M. (*p < 0.05, **p < 0.01, ***p < 0.001; two-way ANOVA followed by Fisher’s LSD post hoc test). n = 3–6/group. Illustration of horizontal section 4 of the mouse brain is originated from the website of http://www.mbl.org, (PFC region is highlighted)^69^.

Alterations in gene expression might accompany, or convey long term adaptive changes in behavior and it is known that genetic deficiency of P2X7R have a strong impact on the mRNA expression of a number of genes showing biological plausibility in psychiatric disorders^7^. In the next part of the study we have examined, whether gene expression changes driven by PCP are also subject to regulation by P2X7 receptors. At first, mRNA expression levels of a set of genes encoding ionotropic NMDA (Grin1, Grin2a, Grin2b) and metabotropic (Grm3) glutamate receptors, were examined in prefrontal cortex samples derived from juvenile (18 days old) and young adult (56 days old), P2rx7+/+ and P2rx7−/− mice treated by saline (0.9% NaCl) or PCP (dose of 2 mg/kg) 1 h after the PCP injection. Gene expression was compared to the data found in saline treated, juvenile P2rx7+/+ mice. Whereas we could not detect significant change in mRNA level of these genes in response to either PCP treatment or genotype in the juvenile animals, their expression pattern was substantially altered in the young adult, P2rx7+/+ mice (Fig. 5). Among the NMDA type glutamate receptor subunits, the relative gene expression levels of Grin1 was significantly decreased in PCP treated mice, when compared to saline treated group (Fig. 5a). Although a tendency can be observed, this change did not reach the level of significance in P2rx7−/− mice (Fig. 5a). Interestingly, Grin1 mRNA expression was lower in P2rx7−/− mice, when compared to wild-type counterparts either in the saline and PCP treated groups (Fig. 5a).

As for Grin2a and Grin2b, PCP treatment upregulated their expression in young adult P2rx7+/+ mice (Fig. 5b,c). Once again, these changes were not detected in mice genetically deficient in P2rx7 (Fig. 5b), and turned to into a significant downregulation in case of Grin2b (Fig. 5c). Grin2a and Grin2b levels were also significantly lower in PCP treated P2rx7−/− mice, when compared to PCP treated P2rx7+/+ mice (Fig. 5b,c), whereas in saline treated P2rx7−/− mice Grin2b was upregulated when compared to saline treated P2rx7+/+ mice (Fig. 5c).

mRNA Expression of Grm3 encoding metabotropic glutamate receptor 3 displayed a robust downregulation during adolescence resulting in values close to detection limit in young adult animals, when compared to the juvenile group (Fig. 5d). Therefore data of these groups were also normalized to values of the saline treated, young adult P2rx7+/+ mice (Fig. 5d, inset). PCP treatment caused a significant decrease in the expression level of Grm3, and this change could be observed in the absence of P2X7R as well (Fig. 5d, inset). Genotype did not affect the expression of Grm3 in either groups (Fig. 5d).

**Figure 6:**
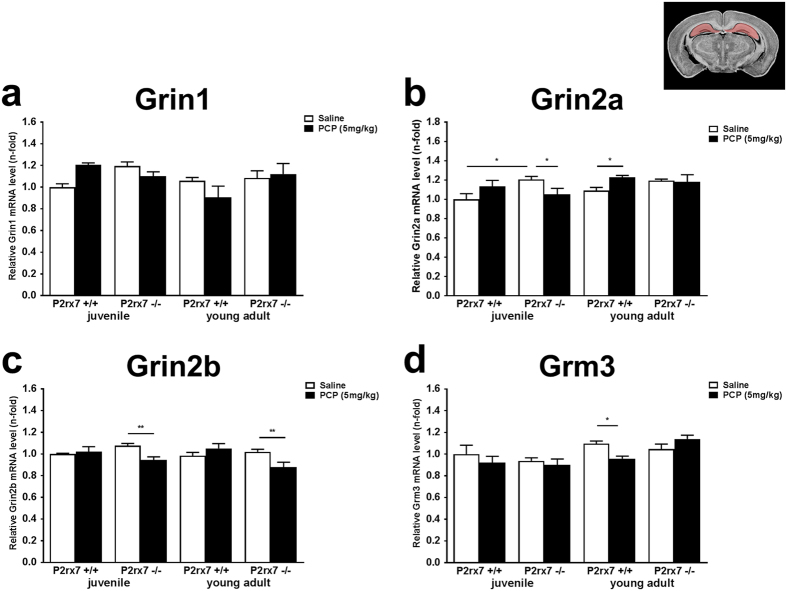
Effect of the higher dose of PCP (5 mg/kg) on the relative mRNA expression levels of ionotropic NMDA (**a**) Grin1, (**b**) Grin2a, (**c**) Grin2b and a metabotropic glutamate receptor, (**d**) Grm3. Samples are derived from HPC of juvenile (18 days old) and young adult (58-60 days old), P2rx7+/+ and P2rx7−/− mice. Gene expression levels of each genes were normalized to the gene expression level of Gapdh. Normalized mRNA expression levels in juvenile saline-treated P2rx7+/+ animals were interpreted as 100%. Data are displayed as the mean ± S.E.M. (*p < 0.05, **p < 0.01; two-way ANOVA followed by Fisher’s LSD post hoc test). n = 3–4/group. Illustration of coronal section 21 of the mouse brain is originated from the website of http://www.mbl.org, (HPC regions is highlighted)^69^.

Because the higher dose of PCP upregulated P2rx7 mRNA in the hippocampus, next we assessed how the expression of the above four genes are altered by genotype and the higher dose of PCP (5 mg/kg i.p.) in the hippocampus of juvenile and young adult mice (Fig. 6). When compared to PFC, changes in the expression of genes were relatively mild in the hippocampus. There was no significant change in Grin1 expression by PCP treatment in juvenile and young adult mice of either genotype (Fig. 6a) and the same holds true for Grin2b (Fig. 6c). A slight, but significant upregulation of Grin2a could be observed in young adult P2rx7+/+ mice, and this change was not detected in P2rx7−/− mice (Fig. 6b). In contrast, Grm3 was slightly downregulated by PCP in young adult P2rx7+/+ mice and this effect also disappeared in age-matched P2rx7−/− mice (Fig. 6d).

**Figure 7:**
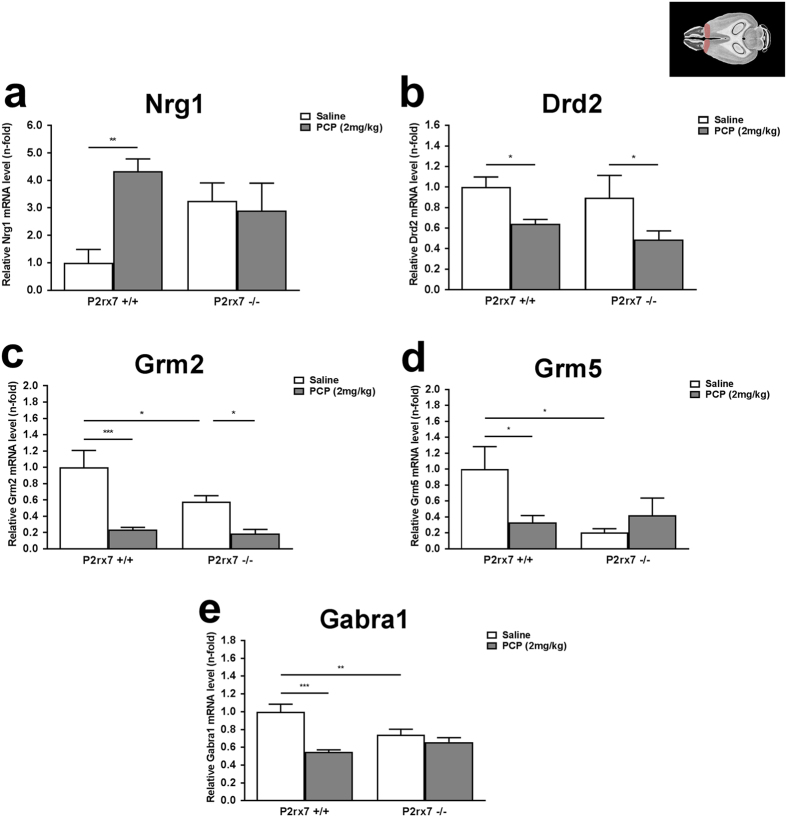
Effect of the lower dose of PCP (2 mg/kg) on the relative mRNA expression levels of selected, schizophrenia related genes, (**a**) Nrg1, (**b**) Drd2, (**c**) Grm2, (**d**) Grm5, (**e**) Gabra1. Samples are derived from PFC of young adult (56 days old), P2rx7+/+ and P2rx7−/− mice. Gene expression levels of each genes were normalized to the gene expression level of Gapdh. Normalized mRNA expression levels in saline-treated P2rx7+/+ animals were interpreted as 100%. Data are displayed as the mean ± S.E.M. (*p < 0.05, **p < 0.01, ***p < 0.001; two-way ANOVA followed by Fisher’s LSD post hoc test). n = 3–6/group. Illustration of horizontal section 4 of the mouse brain is originated from the website of http://www.mbl.org, (PFC region is highlighted)^69^.

Next, we extended gene expression profiling studies to a further selection of genes showing biological plausibility for schizophrenia (Fig. 7). These genes were the following: D1 and D2 dopamine receptors (Drd1, Drd2), catechol-o-methyltransferase (Comt), Neuregulin 1 (Nrg1), metabotropic glutamate receptor subtype 2 and 5 (Grm2, Grm5), GABA_A_ receptor subunit α1 and α5 (Gabra1 and Gabra5). Because the previous experiments showed that gene expression changes by PCP treatment and genotype were more pronounced in the PFC and in the young adult animals, PCP (2 mg/kg i.p.) and genotype induced changes were analyzed in the PFC in this age group. The following alterations were found: a profound upregulation of Neuregulin1 (Nrg1) mRNA expression was found in response to PCP treatment which was absent in P2X7 receptor deficient animals (Fig. 7a). A downregulation of D2 receptor mRNA (Drd2) by PCP was also observed in P2rx7+/+ mice; however this change was persisted in P2rx7−/− mice (Fig. 7b). Likewise, a PCP induced downregulation of metabotropic glutamate receptor subtype 2 (Grm2) was found in the prefrontal cortices of both P2rx7+/+ and P2rx7 −/− mice (Fig. 7c). In contrast, PCP treatment significantly decreased the mRNA expression of metabotropic glutamate receptor subtype 5 (Grm5, (Fig. 7d)) and GABA_A_ receptor subunit α1 (Gabra1, (Fig. 7e)) and these changes were eliminated in the absence of P2X7R. mRNA expression of Grm2, Grm5 and Gabra1 was significantly lower in saline treated P2rx7−/− mice when compared to saline treated P2rx7+/+ mice (Fig. 7c–e). No change in D1 dopamine (Drd1), catechol-o-methyltransferase (Comt) and GABA_A_ receptor subunit α5 (Gabra5) was detected by either PCP or genotype (data not shown).

## Discussion

Schizophrenia is a multidimensional disorder characterized by positive, negative and cognitive symptoms involving a multiplicity of neurotransmitters and signaling pathways. The potential role of purinergic signaling system in schizophrenia has been raised by several studies^13^; however so far only the involvement of A_2A_ adenosine^25^ and P2Y_1_ receptors^26^ have been tested in experimental disease models, respectively.

PCP, as an NMDA-type glutamate receptor antagonist evokes schizophrenia-like symptoms in human^27^, and its application in rodents is a widely applied and reliable pharmacological model to investigate the disease pathophysiology and to test potential new treatments^28^,^29^. PCP-induced schizophrenia models mimic a wide facet of symptoms including positive, negative and cognitive symptoms and the paradigm used in our study replicated the expected behavioral alterations described previously^24^.

The principal new finding of the present study is that both genetic deletion and pharmacological blockade of P2X7Rs lead to significant alterations in behavior induced by PCP in mice and suggesting that these receptors are endogenously activated in this model. Whereas PCP induced hyperlocomotion and stereotype behavior were alleviated by both genetic deletion and pharmacological inhibition of P2X7Rs the basal level of social interactions were increased in P2rx7−/− mice but not by JNJ-47965567 suggesting a developmental effect of genetic deletion. In this respect our findings differ from a previous observation using another brain permeable P2X7R antagonist, JNJ-42253432, which increased social interactions and social preference irrespectively from prior stress exposure in rats^11^. The reason for this discrepancy could be slight differences in experimental protocol, species variance or the distinct affinities of the two P2X7R antagonists to splice variants of P2rx7, responsible for this particular effect^30^,^31^. Nevertheless, JNJ-47965567 restored social withdrawal elicited by PCP.

**Figure 8:**
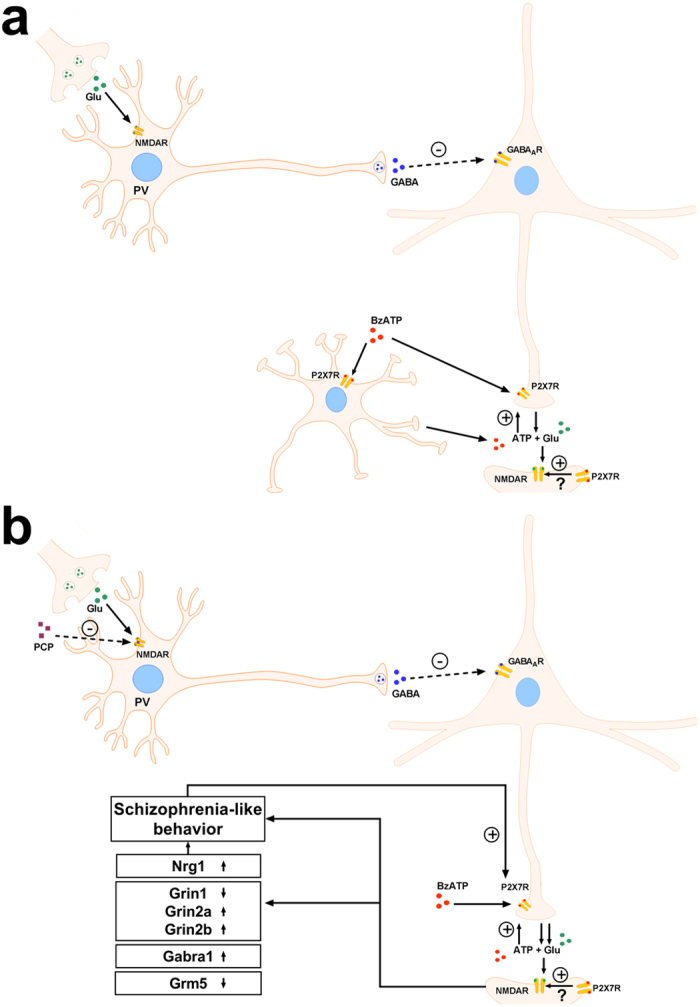
The hypothesis for the potential interactions of the P2X7 receptor with NMDA receptor regulation. (**a**) The stimulation of P2X7Rs by ATP/BzATP increased the release of glutamate in the prefrontal cortex, which could be derived from nerve terminals or astrocytes. This release of glutamate and NMDA-receptor mediated currents were decreased in the absence or under the pharmacological blockade of P2X7 receptors. These data imply that postsynaptic NMDA receptors are subject to modulation by P2X7 receptors directly or indirectly. (**b**) The NMDA receptor antagonist, phencyclidine (PCP) evokes schizophrenia-like behavior, through the disinhibition of parvalbumin (PV) containing GABAergic neurons synapsing onto prefrontocortical pyramidal neurons, resulting in increased EFS-induced glutamate efflux detected in release experiments. In parallel with behavioral changes, the mRNA expression of Neuregulin 1 (Nrg1), different NMDA receptor subunits (Grin1, Grin2a, Grin2b), the GABA_A_ Receptor α1 Subunit (Gabra1) and the metabotropic glutamate receptor 5 (Grm5) are also dysregulated, and all these alterations are subject to regulation by P2X7 receptors. In turn, PCP treatment upregulates and increases the functional responsiveness of P2X7 receptors, resulting in an increased BzATP-induced glutamate efflux. Figure 8 was created by modifying images purchased in the PPT Drawing Toolkits-BIOLOGY Bundle from Motifolio, Inc (http://www.motifolio.com/neuroscience.html).

It is well known that P2X7R activation leads to increased glutamate release from the hippocampal slices^8^,^17^ and from cerebrocortical nerve terminals^19^. Here we extend these results demonstrating P2X7R-mediated glutamate release from acute PFC slices. Moreover, we show that P2X7R mediated glutamate release is upregulated in response to PCP treatment, which could be a consequence of increased expression of P2X7Rs detected in mRNA expression studies. A similar functional upregulation of P2X7R mediated glutamate release were detected in response to *in vitro* ischemia-like conditions^32^ and recently using oxaliplatin induced neuropathic pain model in the cerebral cortex^33^. Because we used acute slices, with an intact neuron-glia network, P2X7R mediated glutamate release could be originated either from nerve terminals or from the neighboring astrocytes as demonstrated in electrophysiological studies^34^. Moreover, glutamate released by P2X7R activation might also act on either neuronal or glial NMDA receptors. However, in a previous study P2rx7 gene deficiency did not affect NMDA evoked currents recorded from *in situ* cortical astroglia^35^. In the present study, the current amplitude of NMDA mediated currents from layer V pyramidal neurons was slightly alleviated by both genetic deletion and pharmacological blockade of P2X7R (Fig. 4c). Bath-applied NMDA induced currents in whole-cell patch clamp experiments are predominantly caused by direct activation of postsynaptic receptors by external NMDA. Therefore, our findings suggest that prefrontocortical postsynaptic NMDA receptors are under the regulation of P2X7Rs at least in juvenile animals. However, the pyramidal neurons were not isolated synaptically in our patch clamp experiments, therefore contribution of presynaptic mechanisms to the observed effect involving presynaptic NMDA receptors cannot be unequivocally excluded. Although both genetic deficiency and pharmacological blockade of P2X7R reduced the current amplitude evoked by 30 μM NMDA, and the concentration-response curves showed a strong tendency of flattening, these effects were not too robust, which is also compatible with an indirect effect (Fig. 8).

The peak ATP concentration in the synaptic cleft is estimated as high as several hundreds of micromole under neuronal activity^36^. Recently, two studies detected extracellular ATP in a behaviorally relevant concentration in the prefrontal cortex *in vivo*^9^,^37^. These findings explain how PFC P2X7Rs could be endogenously activated under the experimental conditions of behavior experiments.

As for gene expression changes, a prominent increase in the relative Grin2b (NR2B) mRNA expression level was detected after PCP treatment, in the PFC of young adult, but not juvenile P2rx7+/+ mice (Fig. 5c). These data are consistent with the elevation of NR2B protein expression to acute PCP treatment in the frontal cortex of the rat brain^38^, and with human post mortem studies, founding increased NR2B subunit mRNA and protein levels in cortical areas of schizophrenic patients^27^,^39^,^40^, but see ref. 41. *In vivo* treatment with NR2B receptor antagonists reproduce some features of schizophrenia-like behavior, such as hyperlocomotion and impaired PPI in rodents^42^,^43^,^44^,^45^, just like the cortical genetic deletion of NR2B in mice^46^, pointing to the determinant role of this subunit of NMDA receptor in shaping of the schizophrenia-like behavioral changes.

However, earlier studies showed that NMDA receptor antagonist treatment leads to the loss of parvalbumin and GAD67 in cortical areas with the involvement of NR2A subunit of the receptor^47^. In our study, Grin2a (NR2A) subunit mRNA expression level was also higher in the PFC of PCP treated young adult P2rx7+/+ mice (Fig. 5b). Collectively, these observations suggest that PCP induced NR2A and NR2B upregulation found in the PFC of young adult rats are compensatory changes due to NMDA receptor hypofunction elicited by PCP treatment. Importantly, PCP induced changes in mRNA expression level of NR2A and NR2B subunits were not detected in P2rx7−/− mice indicating that the effect of PCP on NMDA receptor subunit expression is mediated or modulated by endogenous P2X7R activation.

As for other schizophrenia related genes, an elevated Nrg1 gene expression level was found in the present study in PFC of young adult P2rx7+/+ animals after PCP treatment (Fig. 7a). There is rather strong evidence that one of the major susceptibility genes for schizophrenia is Neuregulin 1 protein coding gene (NRG1) that might be responsible for a fraction of schizophrenia cases^27^,^48^,^49^. In line with our results, upregulation of NRG1 type 1 mRNA and protein was detected in post-mortem dorsolateral PFC tissues derived from schizophrenic patients^50^,^51^,^52^. Schizophrenic-like phenotype is also detected in heterozygous TM-domain NRG1 mutant mice^53^.

NRG1 signaling has a prominent role in early neural development. NRG1 acts predominantly through the ErbB4 tyrosine kinase receptor and may alter the NMDA receptor levels and their function, by phosphorylation of NR2 subunits of the receptor^54^. However, it is also possible that primary dysfunction of other genes and signaling molecules, leads to the secondary alteration of NRG1 expression and functioning in schizophrenia. Supporting this latter theory and our data Feng *et al*. found that the NMDA receptor antagonist MK801 upregulated NRG1 protein in the PFC of adult rats^55^. Perinatal PCP treatment also caused similar changes increasing NRG1 protein expression in the PFC of adult, but not adolescent rats^56^,^57^. Once again, PCP-induced upregulation of NRG1 mRNA in the PFC was not detected in in P2rx7−/−mice suggesting the specific interaction between P2X7R mediated signaling and NRG1-signaling, possibly indirectly, with the involvement of glutamatergic transmission.

According to current hypotheses, PCP induced NMDA receptor hypofunction leads to a deficit of parvalbumin positive GABAergic interneurons and the consequent cortical disinhibition is responsible for schizophrenia specific symptoms^58^,^59^,^60^. Accordingly, significant decrease in the gene expression level of GABA_A_ receptor subunit α1 was observed after the lower dose of PCP treatment in our experiments, in the PFC of young adult, P2rx7+/+ mice and this change was also eliminated in P2rx7−/− mice (Fig. 7e). Likewise, significant reduction in GABA_A_ receptor subunit α1 mRNA level was observed in cerebral cortices and hippocampus after a single PCP injection in rats^61^.

In contrast to glutamatergic signaling, PCP induced downregulation of D2 receptors and [^3^H]dopamine release from the PFC were not subject to modulation by P2X7 receptors.

P2X7R antagonists have been proposed as a potential drug target in a variety of inflammatory and CNS diseases^3^,^4^. Although first clinical trials with P2X7R antagonists have not proven their efficacy in systemic inflammatory disorders^62^, recent trials proved to be more promising in terms of efficacy and all those compounds, which entered to clinical trials displayed a beneficial risk profile^63^. Moreover, in the past years various classes of small molecule, drug-like P2X7R inhibitors have been developed, which readily enter the brain and display high degree of target engagement in the CNS^64^,^65^. Although further studies should elucidate the role of P2X7R in cortical development and its contribution to schizophrenia endophenotype, our findings points to its role as a potential target in schizophrenia.

## Materials and Methods

### Animals

All studies were conducted in accordance with the principles and procedures outlined in the NIH Guide for the Care and Use of Laboratory Animals and were approved by the local Animal Care Committee of the Institute of Experimental Medicine (Budapest, Hungary, ref. No. PEI/001/778–6/2015). Young adult, 2–3 month old drug and test naïve male wild-type (P2rx7+/+ ) and P2X7 receptor knockout (P2rx7−/−) mice were housed in a light- (12 h on, 12 h off) and temperature-controlled room with food and water available ad libitum. All experiments were performed during the light phase between 7:30 am and 3:30 pm. Homozygous P2X7 receptor P2rx7+/+ mice were bred on a background of C57Bl/6J. The original breeding pairs of P2rx7−/− mice were kindly supplied by Christopher Gabel from Pfizer, Inc. (Groton CT, USA). The animals contained the DNA construct P2X7-F1 (5′-CGGCGTGCGTTTTGACATCCT-3′) and P2X7-R2 (5′-AGGGCCCTGCGGTTCTC-3′), previously shown to delete the P2X7 receptor^66^. Offspring of this mouse line were cross-bred with P2rx7+/+ mice, and the resulting heterozygotes were used as breeding stock for the F1 generation offspring employed in the behavior studies. Genomic DNA was isolated from the tails of P2rx7+/+ and P2rx7−/− animals, and the genotypes were confirmed by PCR analysis.

### Behavior studies

Animals were randomly assigned to different treatment groups with a sample size of 10–12 and six groups/experiment were formed (2 genotypes/pharmacological treatment, 2 doses of PCP + vehicle). Mice were subjected to single i.p. treatment with the selective P2X7 receptor antagonist JNJ-47965567 (30 mg/kg i.p., donated by Janssen Research & Development, San Diego, USA) or its vehicle (30% β-cyclodextrin, Cydex Pharmaceuticals, Lawrence, USA). 30 min after the treatment, mice were injected i.p. with different doses of phencyclidine (PCP, 1.5–2–5 mg/kg Sigma-Aldrich Kft, Budapest) or its vehicle (saline, 0.9% NaCl) in a 10 ml/kg injection volume. The actual dose selection for the lower dose of PCP (2 vs. 1.5 mg/kg) was based on the evaluation of preliminary experiments and behaviorally equipotent doses were administered in the two series of experiment. Forty-five minutes later, mice were submitted to the social withdrawal test, according to the method of Sams-Dodd^67^,^68^. The test was performed in a dark-grey, circular open field. Two unfamiliar mice receiving the same pharmacological treatment were placed into the open field. Mice were placed at opposite sides of the apparatus. Behavior was recorded for 10 min by means of a video camera placed above the open field.

An experimenter blind to the treatments scored all behaviors on video recordings. An independent experimenter checked scoring and reliability, which was usually above 90%. The following behavioral variables were recorded: distance travelled, stereotype behavior, ataxia and social investigation. Phencyclidine-induced stereotyped behavior and ataxia were scored according to the protocol described in^67^. Social investigations were defined as sniffing directed towards the partner, when the nose of the scored mouse touched (or was very close to) the body of the partner. Line crossings and social interactions were recorded for the whole duration of the test by means of a computer-based event recorder.

### [^3^H]Glutamate ([^3^H]Glu)/[^3^H]dopamine ([3H]DA) release experiments

Experiments were performed on young adult (2–3 months) male wild-type (P2rx7+/+ ) and P2X7 receptor knockout (P2rx7−/−) mice, subjected to saline or PCP (2 mg/kg i.p.) treatment 60 min before the experiment. The [^3^H]Glu release experiments were conducted using the method with slight modifications described in our previous papers (e.g.^8^). Briefly, the mice were anaesthetized under light CO_2_ inhalation, and subsequently decapitated. The prefrontal cortex was dissected in ice-cold Krebs solution saturated with 95% O_2_ and 5% CO_2_, sectioned (400-μm-thick slices) using a McIlwain tissue chopper and incubated in 1 ml of modified Krebs solution (113 mM NaCl, 4.7 mM KCl, 2.5 mM CaCl_2_, 1.2 mM KH_2_PO_4_, 1.2 mM MgSO_4_, 25.0 mM NaHCO_3_, and 11.5 mM glucose), pH 7.4, in the presence of 5 μCi/ml [^3^H]glutamic acid ([^3^H]Glu, 9.8 × 10^−8^ M, specific activity 60 Ci/mmol; ARC, Saint Louis, MO, USA) or in some experiments with [^3^H]dopamine ([^3^H]DA, specific activity 60 Ci/mmol; ARC, Saint Louis, MO, USA) for 45 min. The medium was bubbled with 95% O_2_ and 5% CO_2_ and maintained at 32 °C ([^3^H]DA: 37 °C). After loading, the slices were continuously superfused with 95% O_2_ and 5% CO_2_-saturated modified Krebs solution (flow rate: 0.7 ml/min). After a 90 min washout period to remove excess radioactivity, perfusate samples were collected over 3 min periods and assayed for tritium content. The temperature was strictly kept at room temperature (22–23 °C, [^3^H]DA: 37 °C). At 6 min after the start of the collection, the slices were subjected to a 3 min perfusion of the P2rx7 agonist 3′-O-(4-benzoyl-benzoyl)adenosine 5′-triphosphate, (BzATP, 100 μM, Sigma) under Mg^2+^ free conditions and then changed to normal Krebs solution until the end of the collection period or challenged by electrical field stimulation (EFS, 10 Hz, 1 msec).

The radioactivity released from the preparations was measured using a Packard 1900 Tricarb liquid scintillation spectrometer, using Ultima Gold Scintillation cocktail. The release of tritium was expressed as a percentage of the amount of radioactivity in the tissue at the sample collection time (fractional release). The tritium uptake in the tissue was determined as the sum of release + the tissue content after the experiment and expressed in Bq/g. For the evaluation of the basal tritium outflow the fractional release measured in two consecutive 3 min samples under drug free conditions were taken into account. The BzATP/EFS-induced [^3^H]Glu efflux calculated as the net release in response to the respective stimulus by subtracting the release before the stimulation from the values measured after stimulation. HPLC analyses performed in previous studies (ref. 8) revealed that the majority of tritium released by P2X7R activation represents [^3^H]glutamate.

### Electrophysiological studies

#### Brain slice preparation

Young P2rx7+/+ and P2rx7−/− mice pups (16–20 days old) were decapitated and their brains were quickly removed and submerged in ice-cold artificial cerebrospinal fluid (aCSF) saturated with 95% O_2_ and 5% CO_2_ of the following composition (mM): NaCl 126, KCl 2.5, NaH_2_PO_4_ 1.2, CaCl_2_ 2.4, MgCl_2_ 1.3, NaHCO_3_ 25 and glucose 11; pH 7.4. Thin coronal slices (200 μm thickness) were cut from a block of tissue containing the prelimbic portion of the medial prefrontal cortex using a vibrating blade microtome. After being sectioned, 6–8 slices obtained from a single brain were transferred to a holding chamber and stored in oxygenated aCSF at 36 °C for 1 h, and then at room temperature (22–24 °C). Before use, single slices were transferred to a recording chamber (300–400 μl volume) and continuously superfused (3 ml/min) with oxygenated aCSF at room temperature. The bath solution differed from aCSF used for incubation, in that Mg^2 + ^was omitted. The slices were left to recover for at least 15 min before the start of individual experiments. Only one cell was measured in each brain slice.

#### Whole-cell patch-clamp recordings in brain slices

Pyramidal cells in layer V of the prefrontal cortex were visualized with an upright microscope equipped with a × 40 water immersion objective (Axioscope FS; Carl Zeiss). Patch pipettes prepared from borosilicate glass capillaries were filled with intracellular solution of the following composition (mM): potassium gluconate 140, NaCl 10, MgCl_2_ 1, HEPES 10, EGTA 11, Mg-ATP 1.5, Li-GTP 0.3; pH 7.3 adjusted with KOH solution. Pipette resistances were in the range of 5–7 MΩ. After establishing whole cell access, the system was left for 5–10 min to allow for the settling of diffusion equilibrium between the patch pipette and the cell interior. Current were registered at a holding potential of −70 mV, in the voltage-clamp mode of the patch-clamp amplifier (Axopatch 200B; Molecular Devices).

Different drugs were applied by changing the superfusion medium. In order to construct concentration-response curves, NMDA (1–1000 μM) was used as an agonist. NMDA was applied for 1.5 min, in increasing concentrations, applications being separated by a superfusion period of 10 min with drug-free aCSF. When the effects of the P2X7 antagonist were investigated, it was present in the superfusion medium throughout the experiment.

Data were filtered at 2 kHz with the inbuilt filter of Axopatch 200B, digitized at 5 kHz, and stored on a laboratory computer using a Digidata 1200 interface and pClamp 10.0 software (Molecular Devices).

### Gene expression analysis

To examine the age-related mRNA expression levels of P2X7 receptor in the prefrontal cortex (PFC) and hippocampus (HPC) 4, 18, 35 and 56 days old naive P2rx7+/+ mice (8 animals/group) were used. To investigate the effect of PCP on the P2X7 receptor gene expression level in young adult (56 days old) mice were given an intraperitoneal (i.p.) injection of sterile saline (0.9% NaCl) or phencyclidine (PCP, 2 and 5 mg/kg, Sigma-Aldrich Kft, Budapest). To identify genes involved in PCP-induced changes in the juvenile (18 days old) and young adult (56 days old) P2X7 receptor wild type (P2rx7+/+ ) and knockout (P2rx7−/−) mice we used the same treatments that were mentioned above. Approximately 1 hour after the treatment prefrontal cortex (PFC) and hippocampus (HPC) samples were collected from all animals (n = 3–6 mice/group). Total RNA samples were isolated and purified from homogenate using the RNeasy Lipid Tissue Mini Kit (Qiagen) according to the manufacturer’s instructions. To measure the total RNA concentration and the integrity of the RNA samples, the Agilent 2100 Bioanalyzer (Agilent Technologies, Palo Alto, CA) was used with Agilent RNA 6000 Nano Kit (Agilent Technologies, Palo Alto, CA). Tetro cDNA Synthesis Kit (Bioline USA Inc, Taunton, MA) was used according to the manufacturer’s protocol to synthesize the first strand cDNA from the RNA samples. The relative quantification of target genes expression levels were performed by quantitative real-time PCR analysis (ViiA™ 7 Real-Time PCR System, Applied Biosystems and Life Technologies, Foster City, CA, USA).

### Real-time PCR

TaqMan® Fast Universal PCR Master Mix (2×), No AmpErase® UNG with TaqMan® Gene Expression Assays (Applied Biosystems and Life Technologies, Foster City, CA, USA) were applied in real-time PCR experiments, according to the manufacturer’s instructions. In comparison to the age-related mRNA expression levels of P2X7 receptor, the normalized P2rx7 mRNA expression level of 4 days old animals were interpreted as 100%. To examine the effect of PCP on the P2X7 receptor gene expression level in two different doses (2 mg/kg or 5 mg/kg), the normalized P2rx7 mRNA expression level in the PFC of the saline-treated group were interpreted as 100%. Four types of glutamate receptor gene expression levels (Grin1 (NR1), Grin2a (NR2A), Grin2b (NR2B), Grm3 (mGluR3)) were analyzed after the PCP treatment, in P2rx7+/+ and P2rx7−/−, juvenile (18 days old) and young adult (56 days old) mice PFC (PCP dose of 2 mg/kg), and HPC (PCP dose of 5 mg/kg). The gene expression levels of the juvenile, saline-treated P2rx7+/+ animals were interpreted as 100%. The effect of lower dose of PCP (2 mg/kg) on the relative mRNA expression levels of selected, schizophrenia related genes, (Nrg1, Drd2, Grm2, Grm5, Gabra1) were measured in PFC samples of young adult (56 days old), P2rx7+/+ and P2rx7−/− mice. The gene expression levels in the P2rx7+/+ and saline-treated animals were interpreted as 100%. All of the gene expression levels were normalized to the mRNA expression level of the glyceraldehyde 3-phosphate dehydrogenase (Gapdh) as an endogenous control (housekeeping gene). The list of the TaqMan® Gene Expression Assay ID of the target genes can be found as Supplementary Table S1.

### Data analysis and statistics

For the statistical analysis of gene expression and behavior data, STATISTICA 64 software (StatSoft. Inc., Tulsa, OK, USA) was applied. Differences were analyzed by Kruskal–Wallis ANOVA (behavior studies) and two-way ANOVA followed by Fisher’s least significant difference (LSD) post hoc test (gene expression studies). Respective data derived from release experiments were analyzed by Student t-test (pairwise comparisons) or one-way ANOVA followed by Tukey test (multiple comparisons), as appropriate. For the electrophysiology, data were analyzed off-line using pClamp 10.0 software (Molecular Devices). Concentration–response curves for NMDA were fitted using the logistic function of SigmaPlot (Systat). Two-way ANOVA was used for statistical analysis. All data are expressed as mean ± S.E.M. (*p < 0.05, **p < 0.01, ***p < 0.001). A probability level of 0.05 or less was considered to reflect a statistically significant difference.

## Additional Information

**How to cite this article**: Koványi, B. *et al*. The role of P2X7 receptors in a rodent PCP-induced schizophrenia model. *Sci. Rep.* **6**, 36680; doi: 10.1038/srep36680 (2016).

## Supplementary Material

Supplementary Information
