# Supplementary material for: The role of P2X7 receptors in a rodent PCP-induced schizophrenia model
Source: Sci Rep. 2016 Nov 8;6:36680. doi: 10.1038/srep36680 (PMC5099752; doi:10.1038/srep36680)
Supplement: Supplementary Information [file srep36680-s1.pdf]

**The role of P2X7 receptors in a rodent PCP-induced schizophrenia model**

Bence Koványi, Cecilia Csölle, Stefano Calovi, Adrienn Hanuska, Erzsébet Kató,

László Köles, Anindya Bhattacharya, József Haller and Beáta Sperlágh

**Supplementary Table S1.**

| Gene name | TaqMan® Gene Expression Assay ID |
|---|---|
| P2rx7 | Mm01199500_m1 |
| Grin1 | Mm00433790_m1 |
| Grin2a | Mm00433802_m1 |
| Grin2b | Mm00433820_m1 |
| Grm3 | Mm00725298_m1 |
| Nrg1 | Mm01212130_m1 |
| Drd1 | Mm02620146_s1 |
| Drd2 | Mm00438545_m1 |
| Comt | Mm00514377_m1 |
| Grm2 | Mm01235831_m1 |
| Grm5 | Mm00690332_m1 |
| Gabra1 | Mm00439046_m1 |
| Gabra5 | Mm00621092_m1 |
| Gapdh | Mm99999915_g1 |
